# Transformative advances in Guillain–Barré syndrome research

**DOI:** 10.1128/msphere.00436-26

**Published:** 2026-09-24

**Authors:** Christine M. Szymanski, Ashley M. Rogers, Leandre M. Glendenning, Dee Warmath, Jarrod J. Mousa, Tiffany M. Daugherty, Nadja Zeltner, Jessica L. McAlpine, David R. Tribble, Glen A. Cook, Geert-Jan Boons, Nowshin Papri, Shoma Hayat, Israt Jahan, Zhahirul Islam, Rachel J. Richardson, Charles E. Whitehead-Tillery, Julia A. Bell, Linda S. Mansfield

**Affiliations:** 1 Complex Carbohydrate Research Center, University of Georgia 123423, Athens, Georgia, USA; 2 Department of Microbiology, University of Georgia 189270, Athens, Georgia, USA; 3 College of Family and Consumer Sciences, Biomedical and Translational Sciences Institute, University of Georgia 138574, Athens, Georgia, USA; 4 Department of Biomedical Sciences, College of Medicine, Florida State University 12236, Tallahassee, Florida, USA; 5 Center for Molecular Medicine, University of Georgia 663743, Athens, Georgia, USA; 6 Department of Biochemistry and Molecular Biology, University of Georgia 174518, Athens, Georgia, USA; 7 Department of Cellular Biology, University of Georgia 200747, Athens, Georgia, USA; 8 Infectious Disease Clinical Research Program, Department of Preventive Medicine and Biostatistics, Uniformed Services University of the Health Sciences 231651, Bethesda, Maryland, USA; 9 Department of Neurology, Uniformed Services University of the Health Sciences 369573, Bethesda, Maryland, USA; 10 Department of Neurology, Naval Medical Center Portsmouth 19951, Portsmouth, Virginia, USA; 11 Chemical Biology and Drug Discovery, Utrecht Institute for Pharmaceutical Sciences, and Bijvoet Center for Biomolecular Research, Utrecht University 534214, Utrecht, the Netherlands; 12 Department of Chemistry, University of Georgia 735294, Athens, Georgia, USA; 13 Gut-Brain Axis Laboratory, Infectious Diseases Division, icddr, b, Dhaka, Bangladesh; 14 Comparative Enteric Diseases Laboratory, Large Animal Clinical Sciences, College of Veterinary Medicine, Michigan State University, Food Safety & Toxicology 70726, East Lansing, Michigan, USA; 15 Microbiology, Genetics and Immunology, College of Natural Sciences, Michigan State University, Biomedical and Physical Sciences 3078, East Lansing, Michigan, USA; Virginia-Maryland College of Veterinary Medicine, Blacksburg, Pennsylvania, USA

**Keywords:** antibodies, autoimmune neuropathy, *Campylobacter jejuni*, Guillain–Barré syndrome, lipooligosaccharides, AMAN, AIDP

## Abstract

The Guillain–Barré Syndrome Innovation Alliance held its inaugural meeting focused on creating treatment solutions for Guillain–Barré syndrome (GBS), an autoimmune disease increasingly recognized as a postinfectious neuropathy and the most common cause of paralysis since vaccines for polio. These collaborators, funded by the Department of War, Congressionally Directed Medical Research Programs, and the National Institute of Allergy and Infectious Diseases, span institutes/universities, including the University of Georgia, Michigan State University, Florida State University, the University of Utrecht, the International Centre for Diarrhoeal Disease Research, and the Uniformed Services University of the Health Sciences. Group efforts span biochemistry, epidemiology, immunology, medicine, microbiology, microbial ecology, glycobiology, neurology, neuroscience, pharmacology, and multiomics data science/computational biology. Our team is committed to the advancement of two core objectives: expanding understanding of biological mechanisms of GBS and developing more efficacious, targeted therapies. We are dedicated to finding alternative therapies for patients unresponsive to intravenous immunoglobulin and to creating affordable options to eliminate financial barriers to care.

## INTRODUCTION

In May 2026, a team of collaborators met at Michigan State University (MSU) to provide research highlights from two U.S. federal initiatives. One project, funded by the Department of War Office of the Congressionally Directed Medical Research Programs, Peer Reviewed Medical Research Program (PRMRP) Investigator-Initiated Research Award, was aimed at investigating the “Contribution of the human gut microbiome to the development and severity of Guillain–Barré syndrome (GBS).” The second project, funded by the National Institutes of Health, National Institute of Allergy and Infectious Diseases, Clinical Neuroimmunology and Brain Tumor (CNBT) Section, focused on the “Development of ganglioside specific IgGs for the treatment of GBS.” The researchers spanned institutes and universities from Bangladesh (icddr,b), the Netherlands (University of Utrecht), and the United States, including Florida State University, University of Georgia (Complex Carbohydrate Research Center, Department of Microbiology, Department of Chemistry, Department of Biochemistry and Cellular Biology, College of Family and Consumer Sciences, and Center for Molecular Medicine), and Michigan State University (Department of Large Animal Clinical Sciences, College of Veterinary Medicine, and Department of Microbiology, Genetics & Immunology, College of Natural Sciences). The multidisciplinary teams contributed a broad array of expertise in clinical trials and patient sample collection and assessment; chemical synthesis and glycan array technology; B cell isolation, single-cell sequencing, antibody–ligand biochemical and structural characterization; microbial glycobiology, antibody assessment, and analytical identification; patient questionnaires and survey data comparison in human environments, behavior, and psychology; induced-pluripotent stem cell neuronal differentiation and testing; and microbial ecology analyses, gut–brain axis pathogenesis, innate and adaptive immune pathway assessment, metabolomics, and GBS model development.

## GBS SIGNIFICANCE AND KNOWLEDGE GAPS

GBS is the world’s leading cause of acute neuromuscular paralysis, an acute, inflammatory, demyelinating polyneuropathy characterized by rapidly progressive, symmetric ascending paralysis that begins as weakness and paresthesia (pins and needles/tingling in hands and feet) that progresses to numbness in distal limbs and weakness that can impair gait (1). Injury progresses from distal to proximal muscles and, in severe cases, impacts muscles of respiration so that mechanical ventilation is required. GBS is now considered the most common cause of paralysis since the introduction of vaccines for polio. Reports describe an incidence of 1.3–1.9 GBS cases per 100,000 in the United States annually (1, 2). Approximately 5% of U.S. GBS patients die; 15%–20% do not recover completely; and lifelong disability may result. Postinfectious GBS is common and has been associated with many pathogens, including SARS-CoV-2, influenza virus, Zika virus, cytomegalovirus, Epstein–Barr virus, *Mycoplasma pneumoniae*, and particularly *Campylobacter jejuni* (3). *C. jejuni* causes a high incidence of foodborne disease worldwide (4, 5) and is the most frequently identified antecedent infection to GBS autoimmunity (6). Disease begins 2–3 weeks after *C. jejuni* infection, and onset correlates with peak serum autoantibodies (7). It is clear that *C. jejuni* carriage or colonization is sufficient to trigger GBS. To shield itself from host immunity, *C. jejuni* is capable of mimicking various gangliosides present on nerves, using its surface lipooligosaccharides (LOS) (8). When the host produces antibodies against *C. jejuni* LOS to drive host defense and pathogen clearance, those antibodies also bind to gangliosides, e.g., GM1, GA1, or GD1a on peripheral nerves, due to molecular mimicry. Once these antiganglioside antibodies attack nerves, they trigger the complement cascade terminating in formation of the membrane attack complex, causing demyelination and damage to motor and sensory nerves with subsequent inflammation (9–11). Although *C. jejuni* accounts for the majority of GBS cases (12), very little has been done to determine if members of the gut microbiome contribute to GBS outcomes.

Major gaps exist in understanding GBS polyneuropathy, including determining why only some people with common preceding infections develop the disease, but others do not. Key challenges are numerous, including understanding the mechanisms of the molecular trigger(s), developing faster effective therapies, and addressing the causes of significant global, regional, and subtype variations in disease. Additionally, there are no established, reliable biomarkers to predict who will develop GBS after an infection or who will develop the most severe, life-threatening forms. This makes it particularly difficult for clinicians to predict which patients with incipient paralysis will progress to respiratory paralysis. Additionally, there are treatment limitations with no proven, highly effective treatments beyond plasma exchange (PLEX) and intravenous immunoglobulin (IVIg). Unfortunately, PLEX and IVIg are not cures; they only reduce severity and shorten recovery time. Thus, many patients experience long-term, persistent pain or disability throughout their life.

Access to examining GBS patient populations provides tremendous opportunities for advancing neuroimmunology, understanding antecedent infection triggers, and tracking long-term neurological recovery to improve therapies and patient outcomes. Systematic study of these cohorts offers key benefits to both clinical research and public health, such as epidemiological tracking and prognostic modeling. Yet, effectively mapping disease pathways and developing treatments rely on combining *in vitro* and *in vivo* models with human clinical studies. At this meeting, we discussed significant new tools for this purpose, including human pluripotent stem cell-derived (hPSC) neurons to serve as reliable *in vitro* models (13), blocking antibody technologies to generate novel therapeutics, and mouse models that spontaneously develop peripheral neuropathy after exposure to GBS patient-derived *C. jejuni* isolates to serve as a test bed for therapeutics (14–17).

## GBS HISTORY AND ANTECEDENT INFECTIONS

Dr. Zhahirul Islam began the meeting by providing a historical perspective into the discovery of the autoimmune peripheral neuropathy known as GBS, first described by Guillain, Barré, and Strohl in the early 1900s (18). Currently, treatment for GBS involves IVIg using healthy human pooled IgG. However, clinics report that up to 40% of patients are refractory to treatment (19). Furthermore, the high cost of therapy ($12,000–$16,000 [USD]) renders it inaccessible in low- and middle-income countries, even when available. Dr. Islam’s group is participating in Phase 2/3 clinical trials for GBS treatment, testing an anti-C1q antibody (ANX005) aimed at inhibiting the classical pathway for complement activation (20). These studies are possible because Bangladesh has up to twofold higher rates of GBS per 100,000 population (21) compared to North America (although the U.S. military also reports a higher than average number of cases annually (22, 23).

Our group will publish findings elsewhere comparing GBS patient presentations with structured summaries of patients’ symptoms, history, examination findings, and pathophysiology in Bangladesh in comparison to the U.S. military. The current meeting emphasized how national and international studies are invaluable in better understanding the spectrum of these autoimmune diseases. Our findings cover key GBS variants: acute motor axonal neuropathy (AMAN; most often triggered by *C. jejuni*), acute inflammatory demyelinating polyneuropathy (AIDP; the most common variant in North America), and combined acute motor sensory axonal neuropathy (AMSAN; which causes both motor and sensory dysfunction). This work highlighted that GBS disease is frequently initiated by a preceding infection—most commonly *C. jejuni* in cases of AMAN—where cross-reactive surface carbohydrates stimulate the production of autoantibodies targeting neurons of the peripheral nervous system, and recruiting macrophages and complement, resulting in deposition of membrane attack complexes and subsequent nerve cell damage. *C. jejuni* LOS are notorious for mimicking human gangliosides, particularly GM1. Other infectious agents have also been implicated in triggering GBS, but in our experience, to date, their effects are typically indirect through the induction of hyperimmune responses rather than through specific molecular mimicry mechanisms.

## PRMRP PROJECT WITH HUMAN STUDIES

The PRMRP project included two patient populations. The first group targeted former GBS patients that presented with high antiganglioside antibody titers from a previous GBS study conducted by Drs. Halstead and Islam in 2016 (24). In our study, these GBS patients returned, and samples (including sera, feces, and peripheral blood mononuclear cells [PBMCs]) were compared with samples from *C. jejuni* enteritis patients and with healthy human controls. The methods used by clinicians at the newly created GBS clinic, the sample collection directed by Dr. Islam and his team (Drs. Nowshin Papri, Shoma Hayat, and Israt Jahan), and the extensive patient survey conducted by Dr. Dee Warmath are the subject of a forthcoming paper. However, this preliminary work serves as the foundation for multiple investigations presented at the MSU meeting. Through these and previous studies, this GBS patient cohort has become the best-studied group available to the field. In addition to their survey responses, we plan to collect personal testimonials to further immortalize their GBS stories.

The second patient population investigated in the PRMRP project included testing of sera from military personnel that were deposited in the national serum repository required for mandatory HIV testing. A rigorous screening of military health records conducted by an outside contractor, in collaboration with Drs. David Tribble and Glen Cook, led to the selection of 198 former GBS patients from whom sera are available both before and after GBS diagnosis. These samples were paired 1:2 with healthy staff controls and confirmed *C. jejuni* enteritis patients. Blinded studies are now underway in Dr. Geert-Jan Boons’ lab to assess antiganglioside antibody titers using ganglioside glycan arrays (25) and in Dr. Christine Szymanski’s lab assessing anti-*Campylobacter* antibody responses to later match with patient outcomes.

## ROLE OF GUT MICROBIOME IN GBS DEVELOPMENT AND SEVERITY

An impactful goal of these collaborative projects was to discern how one’s gut microbiome plays a role in susceptibility to autoimmune diseases such as GBS. Important inputs to the gut microbiota driving susceptibility to autoimmunity are dietary patterns, antibiotic usage, pathogen exposure, and environmental toxins. Among these, diet is considered the most significant long-term modifier of the gut microbiota; and a strong association of nutritional status with GBS and the need for dietary recommendations and clinical education in GBS management were shown in a recent report by Biswas et al., who are members of our group (26). Dr. Julia Bell from Dr. Linda Mansfield’s lab at MSU described comparisons of fecal microbial populations from GBS, enteritis, and healthy control patients in the Bangladesh study population and how these microbiota data compared with differences in dietary patterns between the patient groups. Diet and food handling practices affecting GBS risk or health were further expanded in the thorough analyses presented by Dr. Warmath of UGA. Both analyses will lead to publications and follow-up studies examining the role of nutrition in GBS disease susceptibility and recovery, especially through effects on patients’ gut microbial ecology and the nervous system.

Dr. Mansfield spoke about the development and characterization of GBS mouse models (both AMAN and AIDP) that she and her previous trainees have published (14–17). GBS mouse models complement human patient studies to achieve in-depth understanding of disease pathogenesis and pathophysiology and to bridge gaps where human studies are limited by ethical constraints, complex genetic diversity, or inability to track early disease progression. They also facilitate evaluation of therapeutic efficacy, safety, and pharmacokinetics, ultimately accelerating advancements in GBS therapeutics. These mouse models demonstrate many of the same features and neurological sequelae characteristic of human GBS, including inflammation, immune cell recruitment, antiganglioside autoantibodies, changes in gait, nerve demyelination, inflammatory lesions, and respiratory distress. C57BL/6 IL-10^−/−^ mice stably colonized with *C. jejuni* strain 260.94 from a GBS patient developed AMAN-like GBS motor nerve lesions, some of which progressed to AMSAN (17). While *C. jejuni* isolates from human enteritis patients induced type 1/17-cytokine dependent colitis in IL-10^−/−^ mice, isolates from GBS patients colonized these mice without colitis but induced autoantibodies cross-reacting with the sialylated oligosaccharide motifs on the LOS of GBS-associated *C. jejuni* and peripheral nerve gangliosides (17). Infection of these mice with a GBS, but not with a colitis isolate, led to sciatic nerve inflammation and abnormal gait and hind limb movements with character and timing consistent with this syndrome in humans (17). Further work showed that autoantibody responses and associated nerve histologic changes were dependent on IL-4 production by CD4 T cells (16). *C. jejuni* GBS strains significantly enhanced Type 2 mediators IL-4, IL-13, and Gata-3 in the colon and antiganglioside IgG1 antibody responses in plasma. These IgG1 autoantibodies reacted against peripheral nerve gangliosides, GM1 and GD1a.

Mansfield’s group also showed that *C. jejuni* induced autoimmune peripheral neuropathy via the sialoadhesin axis (16). Here, Siglec-1 served as a central antigen-presenting cell receptor mediating uptake of GBS isolates via interaction with the sialylated oligosaccharide motifs found specifically on the LOS of GBS-associated *C. jejuni,* along with ensuing T-cell differentiation and autoantibody elicitation (16). Thus, sialylated oligosaccharide motifs on the LOS of GBS-associated *C. jejuni* acted as both the Siglec-1 ligand for phagocytosis and the epitope for autoimmunity. This work also demonstrated the negative regulatory role of IL-10 in *C. jejuni*-induced autoimmunity. Interestingly, in a subsequent study, the Mansfield lab found that transplanted adult human-derived gut microbiota dominated by *Bacteroidetes* and *Firmicutes* bacterial taxa enhanced type 2 anti-GM1 autoantibody responses following *C. jejuni* 260.94 infection in C57BL/6 wild-type mice (15). *C. jejuni*-specific antibody and autoantibody responses were significantly elevated in infected mice, demonstrating that this human-derived microbiota increased GBS signs even when IL-10 responses were present. Taken together, these studies suggest IL-4 and Siglec-1 blockade as potential therapeutic interventions for GBS (16).

An important contribution to current and future studies was the development of a GBS mouse model derived through fecal transplantation from a Bangladesh patient with recurrent GBS, followed by challenge with *C. jejuni* 260.94, as well as with a bacterial species not previously linked to GBS induction (27). Follow-up studies are currently planned to fully elucidate these novel findings and connect them to results presented by Dr. Leandre Glendenning of Dr. Szymanski’s group, who described experiments performed using an *in vitro* model system of immune tolerance and training (28). These *in vitro* experiments showed unexpected immunological changes with the same microbe used in Mansfield’s transplanted mouse model.

## DEVELOPMENT OF GANGLIOSIDE SPECIFIC IgGs FOR GBS TREATMENT

Following discussion of the PRMRP project, a senior graduate student, Ms. Ashley Rogers from the Szymanski lab, described unanticipated immune responses discovered during examination of the samples collected by Dr. Islam’s team from the Bangladesh GBS patient population. A notable advantage of studying this group of patients is that serum samples collected at the time of GBS diagnosis were still available, and members of this patient group were eager to return to the clinic for further sampling and assessment up to one decade after GBS diagnosis. It was through the novel findings of Rogers that a new project funded by CNBT emerged to focus on targeted immunotherapy for GBS patients. This project expanded the research team to include Drs. Jarrod Mousa, Nadja Zeltner, and Parastoo Azadi and provided even greater opportunities for collaboration.

## ANTIBODY ISOLATION AND THERAPEUTIC ENGINEERING

Dr. Mousa, an expert in therapeutic antibody production, described methods for memory B-cell isolation from PBMCs and new techniques his lab is developing to target low-concentration autoreactive B cells that recognize carbohydrate antigens. This work was then supplemented with state-of-the-art single-cell sequencing and antibody–antigen characterization. Thereafter, Ms. Rogers described the mass spectrometry methods available at the Complex Carbohydrate Research Center for assessing antibody isotype, subclass, and N-glycan composition with Dr. Azadi’s group. This quantitative comparison was performed using 1 µL of patient sera.

## hPSC-DERIVED HUMAN SENSORY NEURONS FOR DISEASE MODELING

Dr. Zeltner described her model of hPSC peripheral sensory neurons (13) and their use in screening GBS patient antibodies for functional attributes as candidate therapeutics. This model adds to the repertoire of new models accessible to the broader research community, including *in vitro* cell lines used by Dr. Glendenning, hPSC-derived neurons validated by Dr. Zelter’s group, and GBS mouse models optimized for use by Dr. Mansfield’s group.

## CONCLUSIONS AND FUTURE PERSPECTIVES

By the conclusion of this meeting, the extended team was enthusiastic about two goals: further uncovering the biological mechanisms behind Guillain–Barré syndrome and creating better, more equitable treatments: specifically targeted therapies for patients unresponsive to IVIg and affordable options for those facing financial barriers to current care. By connecting research groups with different specialties, both nationally and internationally, this meeting facilitated data sharing among various collaborators, benefiting multiple funded projects. There was also great optimism for sharing the developed methods with other consortia, foundations, and international organizations. Our future goals in this research are to have a broader impact on prevention and treatment of GBS among patient populations as well as to drive innovations in identifying potentially susceptible populations, especially those facing recurrent GBS.

## Data Availability

Data from this article will be made fully available without restriction as specified in previous publications.
